# Menopause experience in First Nations women and initiatives for menopause symptom awareness; a community-based participatory research approach

**DOI:** 10.1186/s12905-021-01303-7

**Published:** 2021-04-26

**Authors:** Beate C. Sydora, Bonny Graham, Richard T. Oster, Sue Ross

**Affiliations:** 1 Department of Obstetrics and Gynecology, Faculty of Medicine and Dentistry, University of Alberta, 626-1 Community Service Centre, Royal Alexandra Hospital, 10240 Kingsway Ave, Edmonton, T5H-3V9 Canada; 2 Maskwacis Health Services, Maskwacis, Alberta Canada; 3 Department of Agricultural, Food and Nutritional Sciences, Faculty of Agricultural, Life and Environmental Sciences, University of Alberta, Edmonton, Canada

**Keywords:** Menopause, Menopause experience, Menopause awareness, Menopause symptoms, First Nations women, Indigenous population, Community-based participatory research, Qualitative research, Questionnaire

## Abstract

**Background:**

Little research has been conducted about menopause in First Nations women. In response to the wishes of Cree women living in Maskwacis, Alberta, to start a dialogue on menopause, we undertook community-based participatory research (CBPR) to explore menopause experience and raise awareness of menopause symptoms in the community.

**Methods:**

The research adhered to the principles of Ownership, Control, Access and Possession (OCAP™) and was guided by the interest of the participating women. Local women (target age 40–65 years) were invited to participate in workshops using word-of-mouth and community posters in health centers. Five research workshops were held in community settings, attended by experienced women’s health researchers and consenting women. The participants guided the informal discussions. They also completed questionnaires which included menopause-related quality of life. The researchers used extensive hand-written field notes to record data; qualitative content analysis was applied to identify themes. Simple descriptive analysis was used for the questionnaire results. The findings were discussed at a community feedback session and laid the basis for further knowledge translation initiatives.

**Results:**

The five workshops included a total of 37, mostly post-menopausal women with 6–11 women/workshop. The main discussion themes were: "experiences of menopause symptoms" including their impact on quality of life; "menopause knowledge prior to their own experience" with most women feeling that they had insufficient information before menopause; "menopause symptom management" which mainly included practical strategies; "impact of menopause on family members" which was of prime concern with uncontrollable mood changes affecting the whole family and sometimes causing matrimonial disharmony. Questionnaire responses corroborated the workshop discussions. Knowledge translation of the research findings produced two information pamphlets specifically for the Maskwacis community: one for husband/partner, the other for women and family members. These pamphlets have been distributed in all areas of the community.

**Conclusion:**

This CBPR project addressed a topic identified by the community as being important. Community members developed informative pamphlets in response to the women’s concern of lack of understanding for menopause symptoms among families. This simple solution has been widely accepted by community members, opening the possibility of wider discussion about menopause.

**Supplementary Information:**

The online version contains supplementary material available at 10.1186/s12905-021-01303-7.

## Background

Menopause is a natural phase in a woman’s aging process and is defined as the cessation of the menstrual cycle [1]. Hormonal changes during the time before and after the final menstrual cycle can lead to a range of symptoms, including sleep disturbances, hot flashes, loss of energy, depression, and mood swings [2, 3] experienced to a greater or lesser extent. These symptoms can be affected by medical and social determinants of health such as underlying health problems, education, socioeconomic status, and cultural background [4–7].

Indigenous people commonly hold a holistic view of health that goes beyond the biomedical perspective and includes physical, mental, emotional, and spiritual well-being [8]. For some Indigenous people, health management is guided by cultural and traditional practices [9–11]. Further, many Indigenous people live in remote or rural areas, where there may be limited access to health care providers, pharmaceuticals, and specialized clinics compared to those living in urban settings.

Menopause has received little attention in Indigenous health research [12, 13]. Menopause experience, which can include onset, symptom perception, and management varies widely among Indigenous women, depending on biological factors, living conditions, degree of prior menopause-related knowledge, and cultural attitudes to physical life changes [13]. There are still gaps in understanding how cultural differences can shape the menopause experience, considering the impact menopause symptoms can have on quality of life (QOL) not only for the affected women but also for their spouses and other family members.

In response to the wish of women to start a dialogue on menopause issues, we undertook preliminary exploratory research with First Nations women living in the community of Maskwacis, Alberta. The community and Maskwacis Health Services has long-established research relationships with the University of Alberta, including such health topics as diabetes [14] and maternal health care [15]. We were able to build on those established trusting relationships to develop and undertake this menopause research.

The community of Maskwacis, which includes the hamlet of Pigeon Lake, is situated 90 km south of Edmonton and includes four distinct Cree First Nation Bands: Ermineskin Cree Nation, Samson Cree Nation, Louis Bull First Nation, and Montana First Nation. Health care is provided throughout the community (population 19,000) by Maskwacis Health Services, with health and wellness clinics in each Band community and Pigeon Lake, taking a more holistic approach to health and wellness in addressing the unique social, cultural, and health needs of the community [16].

Our preliminary community-based participatory research (CBPR) goals were to engage with women community members to explore in a series of workshops their perceptions and experiences of menopause and the impact of menopause on their QOL. The subsequent knowledge translation efforts responded to the findings of the initial research with the goal of benefiting community members by raising awareness about menopause symptoms and experience.

## Methods

### Study design

We employed a CBPR approach to design and undertake this exploratory research, responding to Maskwacis women's desire to raise awareness of menopause symptoms and their impact on QOL. Indigenous CBPR principles acknowledge Indigenous ways of knowing and assure that data and outcomes are interpreted in a culturally sensitive and contextual appropriate manner [17]. Our research engaged women in menopause workshops to explore their own menopause experiences, incorporating both qualitative methods to record and interpret their personal perceptions, and quantitative methods to describe the circumstances of their lives and explore the impact of menopause on their QOL.

### Advisory committee

An Advisory Committee was established early in the development of the research process. The Advisory Committee involved community Elders, Maskwacis Health Services staff (BG), and University of Alberta researchers (BCS, RTO, SR), who devised a research agreement setting out the collaborative principles according to published ratified guidelines such as the First Nations Information Government Center’s Ownership, Control, Access, and Possession (OCAP™) Principles [18]. The Advisory Committee reviewed research findings from workshops and collectively decided on each step of the research process to assure that the research proceeded in a culturally respectful, relevant, responsive, equitable and reciprocal manner. The Advisory Committee also provided input to the development of further menopause awareness initiatives as the workshop participants wished to extend knowledge of menopause and understanding of its symptoms to spouses and family members.

### Ethics

The study was approved by the University of Alberta Health Research Ethics Board (Pro00051347), which adheres to the recommendations of the Tri-Council Policy Statement (TCPS)-2 chapter 9: Research Involving the First Nations, Inuit and Métis Peoples of Canada [19, 20]. Historically, research was done "on" Indigenous populations, and the results were published with little or no benefit to communities and often portraying Indigenous peoples in an unfavorable light. The TCPS guidelines and OCAP Principles help ensure that research is developed in close collaboration with communities, and that the data and findings are used first and foremost for the benefit of those communities [18–20]. These principles guided the development and conduct of our research, and ensured that the community was involved in leading the local dissemination of the findings before a paper was written for publication.

### Participant recruitment

Our research aimed to involve women in menopause transition age (40–65) from any of the four Nations who were interested in sharing their experiences of menopause with other women. Recruitment posters were displayed in health centers in Maskwacis and Pigeon Lake. Women were also recruited by a local hired research assistant for the first two workshops; subsequent recruitment occurred through word of mouth. All research participants provided written informed consent before joining a workshop.

### Menopause workshops

Menopause research workshops were held in a variety of community venues with only consenting participants and two or three experienced women's health researchers present (BCS, SR ± RO). Workshops were conducted in the English language. BCS provided an introduction about menopause and the women were invited to share their own experiences of menopause, including symptoms and techniques they used to manage them. The discussions were informal and interactive with participants guiding the direction of the conversation. To ensure that issues of interest for menopause research (symptom experience, severity, impact on QOL, management options, etc.) were included, a focus group discussion guide was developed for this project (the focus group discussion guide is provided as Additional file 1). Participants were then provided with a questionnaire including demographics, gynecology and general medical history, lifestyle questions, questions about their perception of menopause and current management, and the validated menopause-specific quality of life (MENQOL) questionnaire [21–23]. Lunch was provided, as well as a $25 gift card for each participant.

### Data collection and analysis

#### Workshop discussions

Extensive hand-written field notes were used to record the workshop discussions as a less intrusive method compared to audio recording. Notes were collected anonymously by the attending researchers who were experienced in this method. After each workshop, the researchers' notes were compared to ensure that all relevant comments were included, and that any interpretation accurately reflected the discussion. Because the discussions were led by the women's own experience, the discussions covered a broad range of topics associated with menopause. The collated notes of each workshop were used as the dataset for qualitative content analysis; themes emerged from the data and were reviewed and discussed with the Advisory Committee. Feedback was provided by participants in follow-up meetings.

#### Questionnaire responses

All anonymized questionnaire responses were entered into a secure REDCap (Research Electronic Data Capture) database for cleaning and analysis [24]. REDCap is an electronic data capture tool hosted and supported by the Women and Children's Health Research Institute at the University of Alberta. Simple descriptive statistics were applied to survey data.

The MENQOL questionnaire was used to complement the qualitative data analysis and evaluate the impact of menopause symptoms quantitatively. MENQOL consists of 29 menopause-specific concerns categorized in 4 domains (vasomotor, psychosocial, physical, and sexual) that assess presence or absence of symptoms, and degree of symptom “bother” in the previous week. MENQOL items are converted for analysis using a scale from 1 (symptom not experienced) to 8 (symptom causes maximum "bother"). Mean domain values are calculated by combining the items for each domain [21].

### Feedback of the research findings to the community

After the initial research was complete, a Community Workshop was held to present the research findings to the community. This workshop was attended by the Advisory Committee members and 15 women from the community. The agenda included a presentation of results, menopause information, other group activities and lunch. During the final wrap-up, one of the community members posed the question: "*So, what is next*?". This comment stimulated an open discussion about how the research-generated menopause information should be distributed for the benefit of women and families in Maskwacis. In response to this wish, several Advisory Committee members and community women agreed to form a working group to decide the best way to translate the research knowledge into action for the benefit of the community.

## Results

Five workshops were held between June and November 2015 at community locations in Maskwacis: Samson Cree Management building, Maskwacis Friendship Center, Ermineskin Income Support Center, Pigeon Lake Satellite Health Center, and Louis Bull Satellite Health Center, providing the opportunity for women from all four Nations to participate. A total of 37 women participated in workshops, (6–11 participants per session) lasting between 70 and 90 min. Since the workshops were held at specific community locations, most women participating in a given workshop knew each other. Questionnaire data were available from 34 participants.

Participant characteristics are summarized in Table 1. All women lived on reserve. Among women who completed the questionnaire the majority of women were post-menopausal (absence of menses for > 1 year) (n = 23), six had irregular menses (peri-menopause) and five still experienced regular menses (pre-menopause). Most women had experienced pregnancies and reported a variety of health conditions concomitant with menopause symptoms (Table 1).

**Table 1:** Characteristics of participants from initial 5 menopause workshop

	Participants
	N=34
*Participant characteristics*	
Age at time of workshop, [years (mean±SD)]	53.4 ± 8.9
Native heritage [N (%)]	
Both parents Cree	19 [55.9]
One parent Cree	13 [38.2]
Both parents other native	1 [2.9]
Not reported	1 [2.9]
Employment status [N (%)]	
Work full time	19 [55.9]
Work part time	3 [8.8]
Not in paid employment	9 [26.0]
Not reported	3 [8.8]
Marital status [N (%)]	
Married	16 [47.1]
Widowed	3 [8.8]
Divorced	3 [8.8]
Currently single^a^	12 [35.3]
*Participant lifestyle*	
Sleeping habit [N (%)]	
> 5 h sleep/night	28 [82.4]
≤ 5 h sleep/night	6 [17.6]
Smoking [N (%)]	
Smokers [> 2 cigarettes/day]	15 [44.1]
Non-smokers	17 [50.0]
Not reported	2 [5.9]
Alcohol consumption [N (%)]	
≤ 7 drinks/week	29 [85.3]
> 7 drinks/week	2 [5.9]
Not reported	3 [8.8]
Caffeine consumption [N (%)]	
≤ 4 drinks/day	31 [91.2]
> 4 drinks/day	3 [8.8]
Exercise [N (%)]	
≤ 2 times/week	25 [73.5]
> 2 times/week	9 [26.0]
*Obstetrics and gynecology history*	
Menstrual periods [N (%)]	
Ceased for more than one year	23 [67.6]
Still menstruating (irregular, change to blood flow)	6 [17.6]
Still menstruating (regular)	5 [14.7]
Pregnancies [N (%)]	
Yes (range 2–12) with or w/o live birth (1–10)	27 [79.4]
No	5 [14.7]
Not reported	3 [8.8]
*Medical history*	
Self-reported conditions^b^ [N (%)]	
Cardiovascular disorder	13 [38.2]
Arthritis	10 [29.4]
Diabetes	8 [23.5]
Sleep disorder	8 [23.5]
Mood disorder	7 [20.6]
Gastrointestinal disorder	3 [8.8]
Cancer	1 [2.9]
Asthma	1 [2.9]
None of the above or any other chronic illnesses	8 [23.5]
Surgery (any type)	24 [70.6]
*Menopause therapies and symptom management*	
Self-reported current therapies and management^b^ [N (%)]	
Do not currently use anything in particular to manage symptoms	12 [35.2]
Current therapies and management^b^	
Lifestyle activities for menopause therapy	6 [17.6]
Cultural therapies (Sweats and Native Medicine)	4 [11.8]
Herbs or vitamin supplements	4 [11.8]
Seek advice from friends or relatives	4 [11.8]
Anti-depressants	3 [8.8]
Seek advice from health care workers	1 [2.9]
Menopausal Hormonal Therapy (MHT)	1 [2.9] (vaginal)
Over-the-counter remedies	1 [2.9]
Relaxation therapy	1 [2.9]
Considered management if symptoms become severe and bothersome^b^ [N (%)]	
Seek advice from health care workers	7 [20.6]
Seek advice from friends or relatives	6 [17.6]
Intend to practice cultural forms of relief	2 [5.9]
Consider hormonal therapy	1 [2.4]
Would consider over-the-counter	1 [2.4]
Not sure yet	9 [26.5]
Nothing in particular	4 [14.7]

^a^Might include previous long relationship

^b^Multiple answers possible

### Workshop discussions

1. Menopause symptom experience

Participants talked very openly about their own experience of menopause: their experiences of menopause onset and symptoms varied widely.

The majority of women talked about experiencing menopause symptoms: most could relate to hot flashes and night sweats. Hot flashes were sometimes extreme; one woman described her feeling as “burning inside and out”, while another woman described it as getting hot from the inside “like firecracker”. Sometimes the main symptom was their face flushing very red. Night sweats could also be extreme, causing women to be drenched in sweat. One woman described an occasion when a new nightdress leaked dye into clean sheets—truly “leaving your mark”. Many women complained about hot feet that required cooling off. One said that she could “start a fire” with her feet when walking. Others complained they feel the cold much more now compared to when they were younger indicating the limited window of comfortable temperature commonly experienced during menopause.

Participants described unpredictability in menstrual blood flow which caused embarrassment and anxiety. Blood flow changes could include both decreases, as well as significant increases in flow. In extreme cases, blood could flow “like a waterfall... need to change [menstrual] pads every 30 min”. For some women the variation in flow caused them to worry that it may indicate a more serious cause (e.g. cancer). Heavy bleeding was sometimes experienced in connection with severe pain that required medical interventions including hysterectomy. The impact of hysterectomy (with or without ovaries being removed) and “tied tubes” was of concern; women discussed how these procedures affected menopause symptoms and how they may influence their future health.

Vaginal dryness was experienced by a number of women and could affect their sex drive. Lack of desire for sex was worrying, particularly for women who had previously enjoyed a healthy and fulfilling sex life. They wondered whether and when their previous desires would return, and were concerned because their partner and relationship suffered.

Many participants experienced physical symptoms related to weakness, stiff and achy joints, and sleep deprivation. Insomnia was often associated with night sweats leading to sleep disruptions. Uncontrollable weight gain was also experienced by some women much to their dismay. Other symptoms included changes to skin and hair texture and hair growth where it was not seen before, such as on face, chin or lengthening of eyebrows. These symptoms were not always recognized as being associated with menopause.

Psychological symptoms such as mood swings, anger, depression, and anxiety were also common, unpredictable and worrisome. “Sometimes you cry all the time or get mad about trivial things”. These psychological symptoms and mood swings were a concern, specifically because of their impact on family life and relationships, and were often connected to other life events such as children leaving home and the start of a new phase in family life. One woman described her newly experienced contentment with being alone; another woman mentioned her withdrawal from gathering with people and socializing; one woman mentioned occasional suicidal thoughts.

Some women complained about memory loss and forgetfulness. This prompted a discussion about menopause-specific versus age-related symptoms.

During one workshop, benefits of menopause were discussed. One woman described how activities were less restricted post-menopause. A major constraint before menopause was not being able to take part in ceremonies (sweats, dancing, etc.) while having one's moon time (menstruation) and for four days afterwards.

2. Menopause knowledge prior to own experience

Most participants felt they had not acquired sufficient information about menopause and symptoms before their own experience, because menopause was “not talked about” by their mothers, aunts, grandmothers or by their doctors.

Several women reported that their mothers, when asked directly about menopause symptoms, declared that menopause was not a problem, “*it was really nothing*”, or endured their symptoms stoically. The women discussed whether their mothers were in denial of menopause symptoms, viewed them as the consequences of a natural, invariable stage of their life, or whether they really had forgotten about their symptoms. One woman suggested that in the past, menopause symptoms may not have been as obvious because people were too busy to deal with symptoms, and sweating was usual when women were occupied doing physical work.

Observing other women experiencing menopause has helped some women prepare for their own menopause. Participants remembered occasions when as young girls they were seeing their mothers, a class teacher, an older female friend or relative experiencing mood swings or hot flashes and being drenched in sweat, “*like someone turning on a tap*”, without knowing what was happening at the time. One woman said she felt grateful to have observed others experience these symptoms. A few women mentioned their older sister or a friend as the source of first menopause knowledge.

In one workshop women discussed whether menopause disregard prevents worry about symptoms and symptom awareness. While this hypothesis was considered by some, the majority agreed with another woman’s statement: “it is the other way around: if we know what to expect, then we would worry less”.

3. Menopause symptom management

While going through their own menopause transition, some participants were prepared to deal with symptoms. One woman found that exercise (specifically walking) was very helpful to control mood swings and calm her thoughts during menopause. Strategies for hot flashes included going outside in winter, leaning against cold concrete walls, or walking on cold surfaces to cool feet. Some women stated that they take changes of clothes with them in case they sweat profusely; others were prepared with extra towels for night sweats. An older woman advised younger women: “Don’t think about it, and it won’t bother you. You can cope well... Open your windows.” For unpredictable menstruation some peri-menopausal women carried extra menstrual products.

Several women knew of others who used traditional herbs and other natural remedies for treatment of menopause symptoms. There were discussions about herbal and root teas used by Elders in the past for a variety of conditions. One woman said that her mother is a healer who knows about treatments for many illnesses. Another woman regretted not learning from her, now deceased, mother-in-law, who was very knowledgeable about plants and traditional Indigenous medicines. Other traditional practices to reduce the impact of menopause were taking good care of feet and not going out with wet hair during menopause. Some women had seen others eating dirt, ash or earth during menopause, perhaps in response to a deficiency that the body was trying to correct.

4. Impact of menopause on family members

A major concern for workshop participants was the effect of severe symptoms on their family relationships, particularly their partner-relationships. It was acknowledged by all that menopause can be a strain on the relationship with a partner. The husband/partner may not understand symptoms such as significant mood swings and decreased libido, leading to matrimonial disharmony. One woman jokingly referenced the impact on male partners: “that’s why it’s called MENopause!” Decline in sex drive was of significant concern for women—they felt this was of major importance to their partners, but could not force themselves to be interested in sex, despite knowing this could adversely impact their relationships and potentially cause partners to think their wives no longer care for them or might be unfaithful. One woman complained that she was sick of always apologizing to her husband for her mood swings.

Participants noted that other family members could also be affected by menopause symptoms. Several women felt they were not understood by their family members, especially because of their unpredictable mood swings, depression and anger. These symptoms could cause strife within the family, particularly if the family members did not understand the cause, or if they were undergoing their own hormonal changes (e.g. puberty). Participants felt that they can only now relate to earlier uncomfortable observations of mother’s, aunt’s or grandmother’s menopausal symptoms. One woman said she has told her children, “Don’t get scared of me, I get scared of myself!”.

Participants felt that, if male partners and family members learned about the effects of menopause, they would be more understanding about changes to women's behavior as they go through the transition. They believed there was a need to educate spouses, children, and family members about menopause symptoms. “This is important. I want my family, my daughters and granddaughters to know what [physical changes are] coming” to prepare family members to deal with apparently inexplicable changes that can occur.

### Questionnaire responses

1. Menopause treatment and symptom management

Questionnaire data revealed that the majority of women were reluctant to use drugs such as hormone therapy or over-the-counter remedies, rather choosing to make lifestyle changes, use cultural therapies or herb and vitamin supplements, or use no specific management option (Table 1). The questionnaire results corroborated the qualitative findings from the workshops that women preferred modest, non-medical strategies. Considering future managements, after participating in the workshops, women were more ready to discuss menopause symptoms with friends or relatives or seek advice from health care professionals (Table 1).

2. Impact of menopause symptoms on QOL

The mean average MENQOL scores ranged from 3.3 (vasomotor domain) to 4.1 (sexual domain) indicating that quality of life on average was negatively impacted during the menopause transition period for workshop participants (Table 2). The MENQOL results also validate the experiences women discussed in the workshops, describing mainly the uncomfortable aspects of menopause.

**Table 2:** Menopause-specific quality of life (MENQOL) outcome for women in eligible age range (40–65 years)

Participants	MENQOL scores
N = 29	[mean ± SD]
MENQOL vasomotor domain^a^	3.3 ± 2.3
MENQOL psychosocial domain^a^	3.8 ± 1.6
MENQOL physical domain^a^	4.0 ± 1.4
MENQOL sexual domain^a^	4.1 ± 2.0

^a^One woman did not complete MENQOL and another completed all sections of MENQOL except the sexual domain

### Knowledge translation

1. Formation of Sohki Tehew group and implementation of menopause awareness strategies

A key qualitative finding was a need for increased knowledge about menopause within the community. Knowledge Translation (KT) was planned by a self-selected group of the two Elders from the Advisory Committee and six women community members with the support of Maskwacis Health Services Director of Community Nursing (BG) and a researcher (SR). This group met four times over the noon hour in Ermineskin Cree Nation to plan the KT strategy; lunch and gift cards were provided. Each meeting had a different purpose:

**Meeting 1:** To discuss future menopause initiatives—the group decided to first develop menopause pamphlets describing symptoms and coping strategies to improve understanding between family members about what women experience during menopause.

**Meeting 2:** To develop separate pamphlets for men and women—a preliminary men's pamphlet was distributed at a men's health conference 13 days later.

**Meeting 3:** To finalize the wording for the two pamphlets—and agree on a Cree language name for the group, the "Sohki Tehew (Strong Heart) Group".

**Meeting 4:** To plan a walk and barbecue event for World Menopause Day (October 18). Unfortunately, this event was cancelled shortly before the scheduled date because the venue was needed urgently for another purpose. (The event was eventually held a year later.)

2. Development of two pamphlets

Two culturally-sensitive pamphlets were developed by the Sohki Tehew Group for use within the community, based on the findings of the qualitative study.

The wording and layout for both pamphlets are very similar, but targeted at different audiences: one for husband/partner, the other for women and other family members (Additional files 2, 3: Appendix 1 -  Menopause information for men and Appendix 2 - Menopause information for women and families). Several sections are designed to help understand about the "Change of Life" i.e. menopause:

1. What are the symptoms of the Change?
2. What actions may help someone during the Change, for example engaging in exercise, talking to an Elder, or taking traditional medicines?
3. What you can do to help someone through the Change?, for example offering support and love?
4. What happens after the Change, when the hormone fluctuations have calmed down?

With these few simple pieces of advice, the goal was to increase understanding and reduce anxiety and stress about the physical and mental changes associated with menopause.

3. Implementation

Following the development of the pamphlets, hundreds of copies have been distributed through Maskwacis Health Services and in the Health Clinics. In addition, the Sohki Tehew Group has taken pamphlets to many community events in the four Nations and Pigeon Lake, including Sohki Tehew Workshops, Powwows, Health Fairs, Indigenous Addictions Week, and Maskwacis Community College and school library events.

## Discussion

Our two-part study involved both exploratory research and KT of the research findings in the communities of Maskwacis and Pigeon Lake. The study provides information on the perception, beliefs, and management strategies for menopause of the First Nations women of Maskwacis and Pigeon Lake, adding to the very limited published work on menopause in Indigenous women in Canada [25]. This study demonstrates the willingness of these women to actively participate in research studies, particularly if it is about a subject such as menopause that will affect them all.

The qualitative and quantitative research from the initial menopause workshops found that women experienced physical and psychosocial symptoms such as vasomotor symptoms, urogenital and sleep issues, and mood swings, impacting their QOL in a manner typical for women in menopause transition stages [26–28]. Few used cultural or other medication to address those symptoms, preferring to use modest physical methods such as cooling strategies to reduce the effect of hot flashes. These choices may be partly due to the loss of a dialogue between generations about female topics as a result of residential schools and westernization that have deprived women of their status [29]. Talking about female issues including menopause is still considered private in many Indigenous communities. An important outcome of this research has been giving women a voice and starting a dialogue to raise awareness and understanding of menopause in the community.

The women’s main concern, however, was menopause-related mood swings, and specifically the effect of their unpredictable and uncharacteristic behavior on their family members, creating strain in family relationships. They identified a need for information to help their family members to understand what they were experiencing during menopause.

A working group of local women went on to develop a KT strategy that would take the research findings directly to the people of Maskwacis and Pigeon Lake. The low-technology solution they developed was pamphlets that provided culturally sensitive information and helpful advice about menopause and have been made freely available throughout the community in the health centers in Maskwacis and Pigeon Lake, and at a variety of community local gatherings not necessarily focused on menopause, ensuring that the research findings can easily be accessed directly by community members. Intervention through educational pamphlets has been shown to increase knowledge and reduce a high level of perceived uncertainty associated with menopause [30]. Knowledge of menopause symptoms and health-impacting problems might help reverse the negative impact that menopause-related health issues impose on family life and relationships [31, 32]. The Maskwacis pamphlets may be useful to help Indigenous women and their families in other areas of Canada.

Our study has several limitations and strengths. The research was conducted in one Indigenous community, and involved only 37 women in the research workshops. The participating women may not be representative of the population of Maskwacis women in menopause transition stages; the workshops may have mostly attracted women with moderate to severe symptoms and menopause-related health issues.

Although the MENQOL questionnaire result correlates with the perceived menopause symptom-related reduced QOL described by the women, this association should be viewed with caution as the MENQOL questionnaire to date has not been specifically validated for use in Indigenous groups.

The small workshop size and community-specific location could be considered a limitation; however, it may also be a strength because the women were very open in their discussions, providing rich information about their experiences, perhaps because they were comfortable with each other. In addition, this was a topic that was clearly of importance to them, and they really wanted to help their families and spouses to understand what was happening as they went through menopause.

A strength of this work was the collaboration between the researchers and community members following TCPS-2 and OCAP Principles [18, 20]. This resulted in a more reflective and therefore, as in our case, prolonged way of conducting research, interacting at each stage with the community. As well, this careful research approach provided the community with sufficient access to the results of the research, while at the same time enabling a broader understanding of the community and women's issues to develop over time.

In addition, this research generated an effective and ongoing Sohki Tehew Group collaboration between the original researchers and Elders and members of the community. Over the five years since the start of the Maskwacis menopause research, the group has gone on to develop grant-funded research about strategies women use to age well, and an intervention designed to increase intergenerational cohesion between young women and older women in the community.

## Conclusions

A series of workshops with First Nations women of the Maskwacis and Pigeon Lake Community on menopause experience identified a need for increasing menopause awareness, particularly for partners and other family members. Guided by CBPR, TCPS-2 guidelines and OCAP principles, a KT initiative was developed. The Sohki Teyhew group consisting of community members, Elders, and researchers was formed to give women a voice to overcome their reluctance to openly discuss menopause, and to implement culturally-appropriate strategies in order to raise awareness and provide education about menopause symptoms for the benefit of the whole community.

## Supplementary Information

**Additional file 1.** Focus group discussion guide.

**Additional file 2.** Menopause information for men.

**Additional file 3.** Menopause information for women and families.

## Data Availability

The data generated or analyzed during this study are available from the corresponding author on reasonable request.

## Abbreviations

CBPR: Community-based participatory research
QOL: Quality of life
OCAP: Ownership, control, access, and possession
TCPS: Tri-Council Policy Statement
MENQOL: Menopause-specific quality of life
REDCap: Research electronic data capture
KT: Knowledge translation
