# Supplementary material for: Menopause experience in First Nations women and initiatives for menopause symptom awareness; a community-based participatory research approach
Source: BMC Womens Health. 2021 Apr 26;21:179. doi: 10.1186/s12905-021-01303-7 (PMC8077762; doi:10.1186/s12905-021-01303-7)
Supplement: Supplementary file 1 — Additional file 1. Focus group discussion guide. [file 12905_2021_1303_MOESM1_ESM.docx]

**Perception of Menopause in First Nation Women of the Maskwacis Community:**

**Focus group discussion guide**

**Welcome**

- Thank for coming and volunteering in the focus group

**Introduce goal of the focus group**

- Learning experience for everyone
- There are no right or wrong answers
- Develop intervention to better manage menopause
- Support understanding for menopause symptoms

**Logistics about the focus groups**

- Duration
- Questionnaire completion at the end of the focus group discussions
- Lunch provided
- Washroom locations

**Consent process**

- Go over consent information to ensure everybody understands their rights, confidentiality, and is clear about the fact that participation is voluntary and that they can stop participation at any time without consequences.
- Explain how and why they were chosen to participate
- State that participants will receive a gift card as a Thank you for spending their time in the focus group

**Introduction of participants and research team**

- It is expected that most participants know each other

**Introduce Menopause**

- Natural stage in a woman’s life
- Definition of menopause
- Basic physiology of menopause
- Hormonal involvement in menopause and symptoms
- Menopause symptoms and risk factors
- Management options

**Discussions general**

- We want discussions to be interactive
- Should be guided by the participants
- Should leave adequate room for discussion of their own experience

**Discussion points to be covered (not necessarily in this order)**

What is your experience of menopause with regard to:

- Age of menopause onset
  - age of onset in relation to their social stage in life
- Knowledge of menopause
  - When did you hear about Menopause first?
  - Where you prepared when you reached menopause age?
- Type of menopause symptoms experienced
  - Consider various categories such prototypic vasomotor symptoms, mental symptoms, physical symptoms, sleep problems and their impacts, urogenital and sexual symptoms
- Perception of menopause and menopause symptoms
  - What were your expectations of symptoms if any?
  - Where symptoms experienced as expected?
  - Discuss severity
- Impact of menopause and its symptoms on quality of life?
  - In which ways do menopause symptoms impact your life?
- Management strategies
  - How do you minimize symptom occurrence?
  - How do you treat symptom severity?
  - Discuss menopause hormone therapy use
- Menopause in the context of cultural believes
  - Is menopause connected to specific beliefs or practices among First Nation women?
  - Do you know of any cultural strategies to treat symptoms?

**Provide participants with questionnaire to complete**

- Answer individual questions
- Mention the option of dropping off the completed questionnaire at the Maskwacis Health Centre

**Conclude Focus group**

- Collect completed questionnaires
- Provide participants with gift cards
- Thanks for coming

**Provide lunch**
