# Supplementary material for: Menopause experience in First Nations women and initiatives for menopause symptom awareness; a community-based participatory research approach
Source: BMC Womens Health. 2021 Apr 26;21:179. doi: 10.1186/s12905-021-01303-7 (PMC8077762; doi:10.1186/s12905-021-01303-7)
Supplement: Supplementary file 2 — Additional file 2. Menopause information for men. [file 12905_2021_1303_MOESM2_ESM.pdf]

Symptoms of the Change

Is your spouse . . .

- Being bad tempered and unpredictable?
- Moody and emotional?
- Having difficulty sleeping?
- Suffering from hot flashes/sweats?
- Always needing the bedroom window open, even in winter?
- Having trouble concentrating?
- No longer interested in physical relations?
- Having irregular moon times?

If so she may be in the Change, even though she has not talked about it

What *IS* the Change?

The Change (**menopause**) is experienced by **ALL** women.

- It occurs naturally at the end of fertility - a woman's sex hormone levels (eg estrogen) become erratic.
- Usually happens between the ages of 45 to 55 (start and end are both unpredictable).
- A year or more after the start, the hormones decrease to a lower level than when she was fertile.
- After the Change, a woman will no longer have moon days, but hot flashes can last 5 years or even longer.
- **Every woman is different** – symptoms can vary from mild to severe, one symptom or many.

(If a woman has surgery to remove her ovaries, symptoms of the Change start almost immediately.)

How can you help your spouse through the Change?

- Talk about what's happening to her.
- Ask her what she is experiencing, how she feels.
- Be sympathetic and understanding.
- Reassure her of your support and love

Find out what she thinks would help her, for example:

- Healthy lifestyle (diet, exercise, regular bedtime, spiritual health).
- Elder counselling.
- Sympathetic listener (family member, friend or professional).
- Hormone treatment (from a doctor) to reduce hot flashes and improve mood.
- Traditional medicines.

What happens after the Change?

- Once the hormones have settled down and she no longer has moon days, a woman will become more even-tempered and have fewer troublesome physical symptoms.
- Hot flashes sometimes continue and dry skin will develop and persist. Memory problems may also continue.

KANEWO ASKAMKAHK (Four Directions)

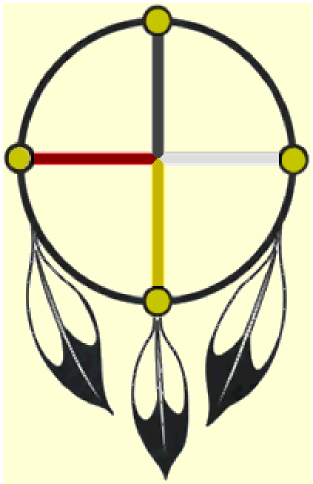

Note: Men's testosterone also decreases as they get older, known as "andropause". The decrease is more gradual than for women, so men don't suffer such extreme changes. Men's changes can be emotional rather than physical.

For further details, please contact Maskwacis Health Services, phone 780-585-2020

This information is provided by:
Maskwacis Women's Menopause Group – ***SOHKI TEYHEW***
Maskwacis Health Services
University of Alberta (Women and Children's Health Research Institute)

Art by: Dawn Marie Marchand

Is your spouse going through "the Change of Life"?

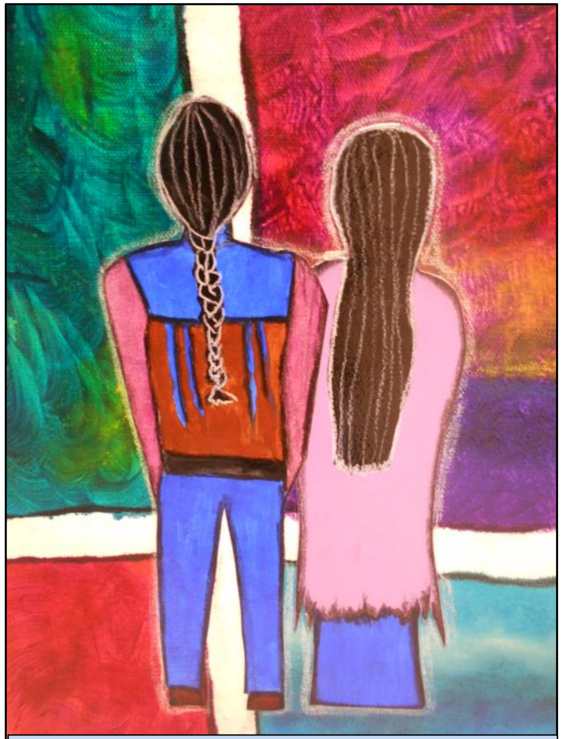

SOHKI TEYHEW (Strong Heart)

Menopause information for men
